# Sustained Yield Forestry in Sweden and Russia: How Does it Correspond to Sustainable Forest Management Policy?

**DOI:** 10.1007/s13280-012-0370-6

**Published:** 2013-03-10

**Authors:** Marine Elbakidze, Kjell Andersson, Per Angelstam, Glen W. Armstrong, Robert Axelsson, Frederik Doyon, Martin Hermansson, Jonas Jacobsson, Yurij Pautov

**Affiliations:** 1 Faculty of Forest Sciences, School for Forest Management, Swedish University of Agricultural Sciences, PO Box 43, 730 91 Skinnskatteberg, Sweden; 2 Department of Renewable Resources, University of Alberta, 751 General Services Building, Edmonton, AB T6G 2H1 Canada; 3 Faculty of Forest Sciences, School for Forest Management, Swedish University of Agricultural Sciences, PO Box 43, 739 21 Skinnskatteberg, Sweden; 4 Institute of Temperate Forest Sciences, Université du Québec en Outaouais, 58 rue Principale, Ripon, QC J0V 1V0 Canada; 5 OOO RusForest Management, 10 Nikolskaya St. Office 502, 109012 Moscow, Russia; 6 JJ Forestry AB, Långgatan 5, 193 30 Sigtuna, Sweden; 7 Silver Taiga Foundation, PO Box 810, 167000 Syktyvkar, Komi Republic Russia

**Keywords:** Silviculture, Annual allowable cut, Sustainable harvest level, Priluzje, Bergslagen

## Abstract

This paper analyzes how sustained yield (SY) forestry is defined and implemented in Sweden and Russia, two countries with different forest-industrial regimes. We first compare definitions of SY forestry in national legislation and policies. Then we study forest management planning in two large forest management units with respect to: delivered forest products and values, how the harvest level of timber is defined, where the harvest takes place, and what treatments are used to sustain desired forest products and values. In Sweden SY forestry is maximum yield based on high-input forest management, and in Russia it is forestry based on natural regeneration with minimum investments in silviculture. We conclude that how SY forestry contributes to SFM depends on the context. Finally, we discuss the consequences of SY forestry as performed in Sweden and Russia related to its ability to support diverse forest functions, as envisioned in sustainable forest management policy.

**Electronic supplementary material:**

The online version of this article (doi:10.1007/s13280-012-0370-6) contains supplementary material, which is available to authorized users.

## Introduction

As early as the eighteenth century the term sustainability was widely elaborated in European forestry as the principle of sustained yield (SY), which later became a general paradigm in forest management world-wide (von Carlowitz 1713; Wiersum 1995; Farrell et al. 2000; Puettmann et al. 2008). The International Union of Forest Research Organizations (IUFRO) defines SY as “the yield that a forest can produce continuously at a given intensity of management, without impairment of the productivity of the land”. Thus, SY management implies continuous production so planned as to achieve, at the earliest practical time, a balance between increment and cutting (Nieuwenhuis 2010).

For the past four centuries SY forestry has been focused mainly on wood for construction, fiber, or fuel. However, the normative interpretation of sustainability in forestry became broader when sustainable forest management (SFM) policies appeared at multiple levels from global to national, and within businesses, at the end of the twentieth century (MCPFE [Ministerial Conference on Protection of Forests in Europe] 1995, 1998, 2001). Today SFM aims at maintaining, now and in the future, sustainable ecological, economic, social, and cultural functions of managed forests through multi-stakeholder participatory approaches (MCPFE 1995, 1998, 2001; Wiersum 1995; Hahn and Knoke 2010). SFM thus encompasses key goals of maintaining the health, integrity, and biodiversity of forest ecosystems; long-term profitability; a healthy environment for local communities; and the cultural identity of forest landscapes (MCPFE 1995, 1998, 2001). This requires that forest managers consider the use of a broad range of ecosystem services through adaptive management and governance in order to be able to handle potentially conflicting demands at multiple spatial scales (Behan 1990; Wiersum 1995; Bawa and Seidler 1998; Farrell et al. 2000; Bouthillier 2001; Hahn and Knoke 2010; Sandström et al. 2011).

At present, society’s interest in sustainable forest management is growing. This is mainly linked to bioenergy production and energy security as well as climate change adaptation and mitigation (Spittlehouse and Stewart 2003). SY forestry is presented as a core principle of forest management that aims at making ecosystems more predictable and reliable for human needs (Davis et al. 2000). Accordingly, forest managers in many countries, particularly those that are part of the Pan-European and Montreal SFM policy processes, claim that SY forestry is an important part of SFM (Korotkov et al. 2009). Others have argued that the timber supply-oriented SY concept is no longer appropriate (Wiersum 1995), and that forest managers need to “develop from being crop managers to ecosystem managers” (Farrell et al. 2000).

Additionally, there are arguments that SY forestry as a single-use management (Behan 1990) focused on wood, changes forest composition and structure, and alters the natural dynamics in forest landscapes (Holling and Meffe 1996; Bawa and Seidler 1998; Luckert and Williamson 2004). As a consequence, forest ecosystems lose native species, habitats, and ecological processes, which affect ecological integrity and resilience (Farrell et al. 2000). There is also a skeptical perception of SY forestry based on arguments related both to the poor rate of success in implementation of the concept in practice, and to increasing demands for diverse ecosystem services from forests, which makes implementation of this concept more difficult (Wiersum 1995; Clapp 1998).

There is a growing understanding of the complex nature of forest ecosystems that acknowledges that a large part of its dynamics is unpredictable, even at short temporal scales (Armstrong 1999; Messier and Puettmann 2011). Any forest management unit is a “coupled human and natural system” (Liu et al. 2007) involving several non-linear ecological, social, and economical interrelationships, organized in hierarchal structures, each acting at different spatial and temporal scales. Recognizing this, Messier and Puettmann (2011) questioned the usefulness of the SY concept as applied until now, and explained its limitations (or obsolescence) for meeting the requirements of the SFM paradigm.

However, different regions have diverse natural, historical, societal, and economical legacies and thus have different starting points in their trajectories of development toward SFM (Lehtinen et al. 2004; Angelstam et al. 2011a). The aim of this paper is to analyze how SY forestry is defined and implemented in countries with different forest-industrial regimes (sensu Lehtinen et al. 2004). Under what conditions is SY forestry an asset or an impediment for SFM implementation? Forest management systems in boreal Sweden and NW Russia are particularly interesting for comparative studies of SY forestry implementation due to their differences in environmental history and societal system (Angelstam et al. 2011a), and similarity in terms of biophysical conditions of forest landscapes (Kuusela 1992; Esseen et al. 1997).

In Sweden about half of the forests are owned by non-industrial private forest owners, and the rest mainly by industrial forest companies, the state and forest commons (The Swedish Forest Agency 2010). The current forest policy gives equal priority to production and environmental objectives (Ministry of Agriculture 2008; Bush 2010). The regulatory parts in the Swedish Forestry Act concern regeneration after final felling, rotation periods as well as considerations related to ecological, social, and cultural values (Ministry of Agriculture 2008). Maximum SY of wood by using even-aged forest management systems is in focus in the current Swedish forest management (Axelsson et al. 2007). Additionally, tree retention is practiced and set-aside areas are managed to maintain ecological, social, and cultural values of forests as providers of post-modern products in terms of tourism and amenity migration (Angelstam et al. 2011a). However, the main end-user is the export-oriented forest industry focusing on value-added production for environmentally concerned markets.

In the Russian Federation, virtually all forest is owned by the state. Forest management is regulated by the Forest Code (FC 2006), numerous sub-laws and governmental regulations. Through state organizations the government defines the harvest level by estimation of the annual allowable cut (AAC) for all state forest management units. The forests are leased by private forest companies, which use the AAC as the upper limit for timber harvest. In the Russian Federation, the Scandinavian model of SY forestry is perceived by industrial forestry stakeholders as the best model for economically profitable forestry (Knize and Romanyuk 2004; Romanyuk et al. 2004). Consequently, there are attempts to introduce this intensive forest management model in the North West (NW) of Russia (Romanyuk et al. 2004). At the same time, NW Russia still hosts large intact forest landscapes (Yaroshenko et al. 2001; Potapov et al. 2008), and there is thus still opportunity to conserve biodiversity at near-natural levels. This is not the case in other parts of the European boreal biome, where biodiversity conservation requires substantial restoration efforts over a long time span (Angelstam et al. 2011b). Intact forest landscapes also offer the opportunity to be used as reference landscapes for conservation in boreal-managed forests in countries with a longer history of forest use (Kneeshaw et al. 2011).

The Russian Federation is a member of the Montreal Process on criteria and indicators for SFM, and both Sweden and the Russian Federation are members of the Pan-European process on criteria and indicators for SFM. The vast majority of boreal forests used for wood production in Sweden and NW Russia are certified according to national Forest Stewardship Council (FSC) standards (Elbakidze et al. 2011).

For this study we used two large forest management units as case studies, one in Sweden and one in the Russian Federation. Both case study areas have similar size, type of forest ecosystems, ownership, and clear ambitions towards SFM. First, we describe how SY forestry is defined in national legislation and policy in Sweden and Russia. Then we compare forest management planning with respect to: (1) delivered forest products and values, (2) how the long-term harvest level of timber is defined, (3) where this harvest takes place, and (4) what treatments of forests are used in order to sustain desired forest products and values. Finally, we discuss the consequences of SY forestry as performed in Sweden and NW Russia, related to the ability of boreal forest landscapes to support ecological, economic, and social–cultural functions, as envisioned in SFM and other sustainability policies.

## Materials and Methods

### Study Areas

We selected as study areas the Bergslagen forest management unit (FMU) of Sveaskog Co. (59°N, 16°E) (hereafter Bergslagen) in Sweden, and the Priluzje state forest management unit (60°N, 49°E) (hereafter Priluzje) in the Russian Federation’s Komi Republic.

Bergslagen is one of Sveaskog’s five FMUs in Sweden. This company is state-owned and has the largest forest area of the four large forest companies in Sweden. Sveaskog manages 13 % of the Swedish productive forests, or forests with mean annual timber production more than 1 m^3^ ha^−1^ year^−1^, and is part-owner of some sawmills. Bergslagen encompasses a total area of 563 629 ha of forest, water and mires, but is fragmented and dispersed over an area exceeding 4 000 000 ha, which is dominated by family-owned forest land and land-owned by other forest companies within nine counties in south-central Sweden. Bergslagen is located in the south and middle boreal ecoregions (Ahti et al. 1968) and has a long history of forest use (Angelstam et al. 2011a). The main tree species are Norway spruce (*Picea abies*) and Scots pine (*Pinus sylvestris*). Forests dominated by birch (*Betula* spp.) and aspen (*Populus tremula*) occupy less than 8 % of the total forested land of Sveaskog. Bergslagen is devoid of naturally dynamic forests, except for small patches of semi-natural forest, many of which are protected. Sveaskog’s forest management was FSC-certified in 1998.

The area of Priluzje is state-owned, covers 810 252 ha, and forms one continuous block. It is located in the south and middle boreal ecoregions of the southwestern part of the Komi Republic in NW Russia. While the same tree species occur in both Priluzje and Bergslagen, forests dominated by birch and aspen occupy almost 40 % of the total forested land in Priluzje. This is a consequence of previous large-scale disturbances by wildfire and logging, and due to a lack of silviculture after harvest. The latter is a result of a very limited road network and of minor investments in silviculture (Elbakidze et al. 2011). Around 60 % of forests in Priluzje are used by 12 private forest and logging companies which hold leases on the forest land for up to 49 years. These companies are responsible for forest management on their leased land. Priluzje still has some pristine forests (12 % of the forest area) with natural dynamics and near-natural composition, structure, and function (CF 2008). In 2003, the forest management conducted by the forest administration in Priluzje became FSC-certified.

### Methods

First, to analyze the SY concept in Sweden and in the Russian Federation, national forest legislation and policies, governmental regulations and forest companies’ documents were reviewed. Second, to analyze delivered main forest products and values, silvicultural treatments performed, harvesting systems and rules in both Bergslagen and Priluzje, a total of ten semi-structured interviews were conducted in 2011. The interviewees were the forest managers of the forest companies operating in the study areas and specialists (from the Swedish University of Agricultural Sciences and the Forestry Research Institute in Sweden and from the Moscow State Forest Institute and the Komi Model Forest in Russia) involved with the development of tools and principles of forest management in the two FMUs. The interview manual was developed to clarify (1) how the level of harvest was defined; (2) what kind of wood assortments were included into the calculation; (3) the planning horizon for a given harvest level; (4) ecological and social considerations related to the calculation of harvest rate; (5) silvicultural treatments of forests in order to sustain desired forest products and values. All interviews were recorded and transcribed. The analysis had a comparative character where differences between the two study areas were identified. Third, we collected data about the location and size of harvest blocks and the volume of harvested wood in the FMUs. In Bergslagen we used the GIS-databases of the Swedish Forest Agency and of Sveaskog to extract those data for the period from 2001 to 2011. In Priluzje those data were available only for the period from 2006 to 2010, and we used the records of the forest leaseholders for each year from the archive of the state FMU. Finally, regarding silvicultural treatments in Bergslagen the GIS-data base of Sveaskog and in Priluzje the archives of the state FMU’s administration were used to extract data about all kinds of forest treatments performed from 2001 to the end of 2010.

## Results

### Delivered Forest Products and Values

#### Sweden-Bergslagen

The main forest products delivered from Bergslagen were sawlogs and pulpwood from Scots pine and Norway spruce, and biomass for bio-fuel production from logging residues (branches and tops) and stumps. The main goal of forest management was to sustain and gradually increase the output of these products from the forests. The consumers of wood from Bergslagen were 17 sawmills, 5 pulp mills, and a few bioenergy producers in neighboring cities.

In order to maintain ecological and socio-cultural forest values, different constraints were incorporated before the strategic forest management plan was done (Table 1). These constraints were stated both in the company’s policy and the Swedish FSC standard (Elbakidze et al. 2011). For example, Sveaskog has an environmental policy from 2003 which states that 20 % of productive forest land at tree, stand and landscape scales in each ecoregion should be used to promote environmental consideration and nature conservation (Sveaskog 2012).

**Table 1:** Constraints in estimation of sustainable harvest level in Bergslagen and in Priluzje

Constraints	Bergslagen	Priluzje
Ecological	Natura 2000 Nature reserve Nature of national interest Classes of forests with biodiversity conservation considerations: NO (all harvests are prohibited), NS (all treatments to create some natural value) and PF (production forest with some small constrains)	Forests with water protective functions along rivers and streams; forests along roads; forests along the spawning places; nature reserves of federal level; forests around settlements Pristine forests, nature reserves of regional level
Socio-cultural	Forests with high social and cultural values for local people (HCVF 5 and 6)	Forests with high social and cultural values for local people (HCVF 5 and 6)

Implementation took place by setting aside areas through Ecological Landscape Planning and introducing modified management practices under the concept of “site-adapted forestry considering nature values” (DeJong et al. 2004). Sveaskog (2012) had established two eco-parks in Bergslagen, where special efforts to provide landscape-scale conservation efforts and socio-cultural considerations were concentrated. Some of the set-aside areas were also formalized through creating legally protected nature reserves for the purpose of conserving biodiversity, areas of national interest with high natural or cultural value, and other valuable natural environments or satisfying the need for outdoor recreation (Naturvårdsverket and Skogsstyrelsen 2005; Angelstam et al. 2011b). The total area of forest stands and landscapes set aside to maintain ecological and socio-cultural functions of forests was 12.1 % of the total forested area in Bergslagen (Elbakidze et al. 2011). Some partial cuttings (including commercial thinning) were performed in the set-aside areas to enhance or restore nature conservation values. At the same time, the general forest management approach was to suppress undesirable natural disturbances such as wildfire and insect outbreaks; but also biodiversity conservation management by burning at least 5 % of the regeneration area on dry and mesic forest land over a 5-year period (FSC 2009).

#### Russia-Priluzje

In Priluzje the main delivered forest products were sawlogs from Scots pine and Norway spruce, pulpwood and firewood mainly from birch and aspen. One large international pulp and paper mill and three regional sawmills were the main consumers of harvested sawlogs and pulpwood. Additionally, forest belts of 1–2 km width around the villages were set aside to satisfy the needs of forest-dependent communities in terms of firewood and non-wood forest products. The forest ecosystem values were maintained by setting aside forests with water and soil protective functions along rivers and streams and along fish spawning areas, and special protected areas with high biodiversity value according to the national nature conservation and forest legislation (Table 1). Those forests were excluded from the calculation of AAC. Additionally, according to the requirements of the Russian national FSC standard, high conservation value forests (HCVFs) with high social and cultural values for local people were defined. The HCVFs which were not protected from commercial use by the national legislation were included to estimation of harvest level for the forested area of Priluzje (FSC 2008). Together forest delivering different forest values other than wood and bio-fuel occupied 21.9 % of total area of forested land (Elbakidze et al. 2011).

### Definition and Estimation of Sustainable Harvest Level

#### Sweden-Bergslagen

In Sweden, the terms “sustainable harvest level”, “allowable cut”, or “harvest level” were used interchangeably to characterize the long-term harvest level estimated in the framework of strategic planning (Eriksson 2008). For example, allowable cut is “the total harvesting volume under sustainable management” (Eriksson 2008). The harvest level included wood volumes originated from both final felling and commercial thinning.

In Bergslagen the continuous wood harvest level was defined by Sveaskog based on the company’s own business and environmental policy, and considering the goals of Swedish forest policy, environmental policy, forest legislation, and the requirements of national FSC standards (Table 2). For determination of the sustainable harvest level, Sveaskog (like all four large forest industry companies in Sweden) used the Forest Management Planning Package (FMPP) developed at the Swedish University of Agricultural Sciences at the end of the 1980s (Jonsson et al. 1993). The FMPP, being a Decision Support System (http://fp0804.emu.ee/), is a set of tools for analyzing the wood resource (Jonsson et al. 1993). The goal of forest management was to find a reasonable compromise between an even flow of wood from the entire FMU and maximum net present value (NPV) based on stand-and-by-stand optimal harvest programs. The FMPP considered forestry focused on timber production (Jonsson et al. 1993).

**Table 2:** Comparison of input forest data, legislation, and regulations to estimation of sustainable harvest level in Bergslagen (Sweden) and Priluzje (Russian Federation)

	Bergslagen	Priluzje
Calculation horizon	100 years	40–60 years
Forest management	Strategic 100 years Tactical 3–5 years Operational 1 year–1 month	Strategic 40–60 years Tactical 10 years Operational 1 year
Basis for estimation of sustainable harvest level	Forest policy Silvicultural policy Environmental policy National forest inventory Timber survey Sample stand inventory Growth and yield models	Forest Code Forest regulations Forest inventory data
Tool for estimation of sustainable harvest level	Software (the Forest Management Planning Package)	Formulas
Collected input data		
Timber-related data	FS: 6 forest assessment areas (size 60 000 ha) (permanent) Fd: register where all forest stands (2–50 ha) are described (site quality, age, diameter, number of stems per ha, species composition) Fs: Forest stands (2–50 ha) (temporal) Fm: Sample plots (5–12) within every sample stand where all individual tree species and diameter are registered, on sample trees also height, quality, and age	FS: 7 local forest management units (average size 116 000 ha) (permanent) Fd: Forest quarters (FQ) (permanent) (total number is 1011, size 800 ha). Within FQ Forest stands (temporal), defined by using air photos (average size 25–30 ha) Fs: No statistical sample—calculation based on aggregated data from all stands Fm: eye-measurement of height, diameter, age of trees, number of stems per ha etc in the field, often in the office using air photos

*FS* forest strata, *Fd* forest description, *Fs* sample as basis for calculation, *Fm* forest measurement

A Geographic Information System (GIS) including a stand register was used to perform hierarchical forest management planning with strategic, tactical, and operational steps. In Bergslagen, the strategic planning horizon for estimation of the harvest level was 100 years (Table 2). The input data for the FMPP was gathered in a special forest inventory designed for acquiring data as a base for FMPP analysis (Jonsson et al. 1993).

Bergslagen was divided into six forest assessment areas, which were considered uniform regarding annual growth and market conditions. Each forest assessment area consisted of a number of forest stands (2–50 ha), which were subject to silvicultural treatments and eventually final felling. Being the most sensitive element in making forecasts of forest development, the growth calculation was based on an individual-tree model simulating growth and mortality (4 % of the growth in forests used for wood production was considered lost due to different natural disturbances) (Jonsson et al. 1993). A suite of sample stands were surveyed for each forest planning unit using approximately ten circular sample plots (Jonsson et al. 1993; Eriksson and Lämås 2001). In each circular plot field measurements of each tree (species and diameter, on sample trees also height, quality, and age) were recorded, site index calculated, nature conservation measures and forest conditions described, and logging and silvicultural costs estimated. In Bergslagen FMU, a total of 450–500 temporary sample plots were used for each forest assessment area. This field inventory is repeated every 8–10 years. Forest yield projections are estimated using single tree growth models that have been verified to be capable of capturing the growth dynamics of boreal forests including growth in mixed species and uneven aged stands (Jonsson et al. 1993).

The average sustainable yearly harvest level of wood in Bergslagen was 1.5 million m^3^ (80 % from final felling and 20 % from commercial thinning), which corresponds 3.4 m^3^ ha^−1^ of forested land, including set-asides. Around 95 % of harvested wood was Norway spruce and Scots pine.

#### Russia-Priluzje

In the Russian Federation the only term used to define how much wood that could be harvested in the long term within a FMU was “annual allowable cut” (AAC). It was defined as “a volume of harvested wood in commercial and protective forests which provides multi-purpose, efficient, continuous and sustainable use of forests, according to the established age of final felling, requirements for biodiversity conservation, maintenance of water protective and protective functions and other benefits of forests” (FAF 2011). The harvest level included wood volumes from final felling and commercial thinning; as well as wood from forest clearing for constructions and other types of activity.

The AAC was estimated by governmental or private forest planning organizations, which were officially appointed by the government to perform this work for different state FMUs (FAF 2011). The calculations of AAC were done according to the Forest Code (FC 2006) and governmental regulations, and considering requirements of the nature conservation legislation (Table 2). The AAC for final felling was estimated as the total area of final felling for each tree species and total allowable volume (m^3^) of harvested wood for each tree species for a given year within a FMU. There were four official methods to calculate the AAC for clearcuts as the total area (ha) of final felling (FAF 2011) (Electronic Supplementary Material, Table S1). The estimation of wood from commercial thinning was done for each FMU according to special governmental instructions.

In NW Russia, including Priluzje, the goal of the strategic plan was to harvest at a level that corresponded to the AAC calculated for the first 10-year period in the strategic plan. Forest operations, including logging and silvicultural treatments, could be conducted by the forest companies only if a 10-year harvest plan was in place and used. In Priluzje the AAC was estimated in two steps. First, the AAC area was defined using the formula of “even forest use” (Table S1). Second, the AAC volume was estimated by multiplying the AAC by area for each tree species by average wood stock per unit area in mature and over-mature forest stands. However, losses of wood due to the different natural disturbances such as forest fires, wind storms, and insect outbreaks, which were common in Priluzje, were not considered in the estimation of the AAC.

The estimation of the AAC was done based on forest inventory data (Table 2). In Priluzje the latest full forest inventory was done in 1992 and updated in 2007. The forests were stratified into seven local forest management units with a total of 1011 forest rectangular blocks of 800 ha each. Each block was then divided into stands with similar age and tree species composition (with average size of 25–30 ha). This was based on the interpretation of air photos from the forest inventory period. The height, diameter, age of trees, number of stems per hectare were measured in the field for the most accessible forest stands, while the description of inaccessible forest stands (which were in majority) were done at the office using air photos and the data from the previous forest inventories.

In Priluzje the AAC was 2.3 million m^3^, or 3.4 m^3^ ha^−1^ of forested land for the period from 2008 to 2017. Approximately 66 % of the AAC was from birch and aspen stands. However, during the last 10 years in reality the forest companies have been harvesting less than 50 % of the AAC (99 % from final felling and 1 % from thinning). The AAC was changed considerably from the previous 10-year period of strategic planning. For example, in 2006 the defined AAC by volume was 1.8 million m^3^ (with 60 % of AAC from birch and aspen). The reason for increasing the AAC was to shorten the rotation period from 100–110 years to 81 years for spruce and pine stands of higher productivity (1–3 classes of site productivity) in 2007.

### Wood Harvest and Forest Treatments

#### Sweden-Bergslagen

**Fig. 1:**
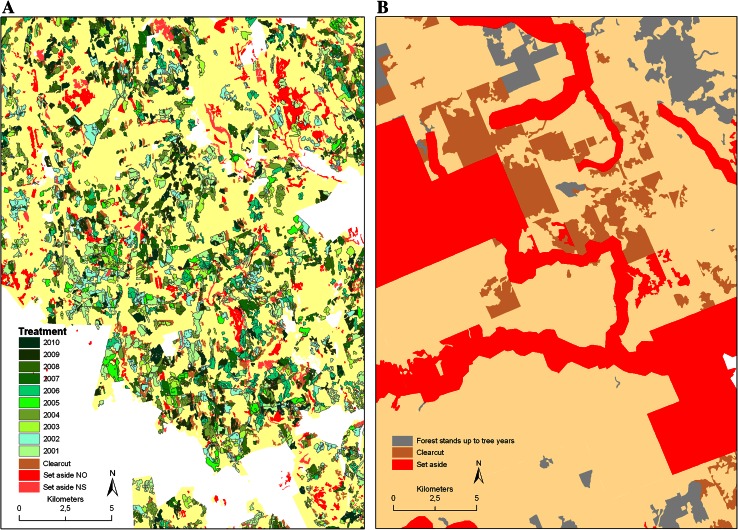
Spatial distribution of forest treatments (see the types of forest treatments in Fig. 2) and clearcuts in Bergslagen (2001–2010) (**A**) and clearcuts in Priluzje (2006–2010) (**B**). The presented maps show more or less representative distribution of those forest operations in the study areas. *Polygons of red color* indicate the stands which are set aside for biodiversity conservation

In Bergslagen final felling was done by clear-felling followed by tree planting or leaving Scots pine seed trees for regeneration on suitable sites. On average 1.3 % of the total area of managed forests excluding set-asides was subject to clear-felling annually (Table 3). The spatial distribution of harvested areas was more or less even across the Bergslagen FMU (Fig. 1).

**Table 3:** Area proportion and number of clearcuts and forest treatments which do not overlap in space performed in Bergslagen (Sweden) and Priluzje (Russian Federation)

Period	Bergslagen	Priluzje
	2001–2010	2006–2010
Mean (range) of area proportion of clearcuts per year (% of total area of forests used for wood production)	1.3 (0.6–1.7)	0.5 (0.4–0.7)
Mean (range) size of individual clearcut (ha)	6.7 (0.3–65.8)	10.6 (0.1–50)
Total area proportion of forest treatments, which do not overlap in space (in % per year)	5.3	0.2

**Fig. 2:**
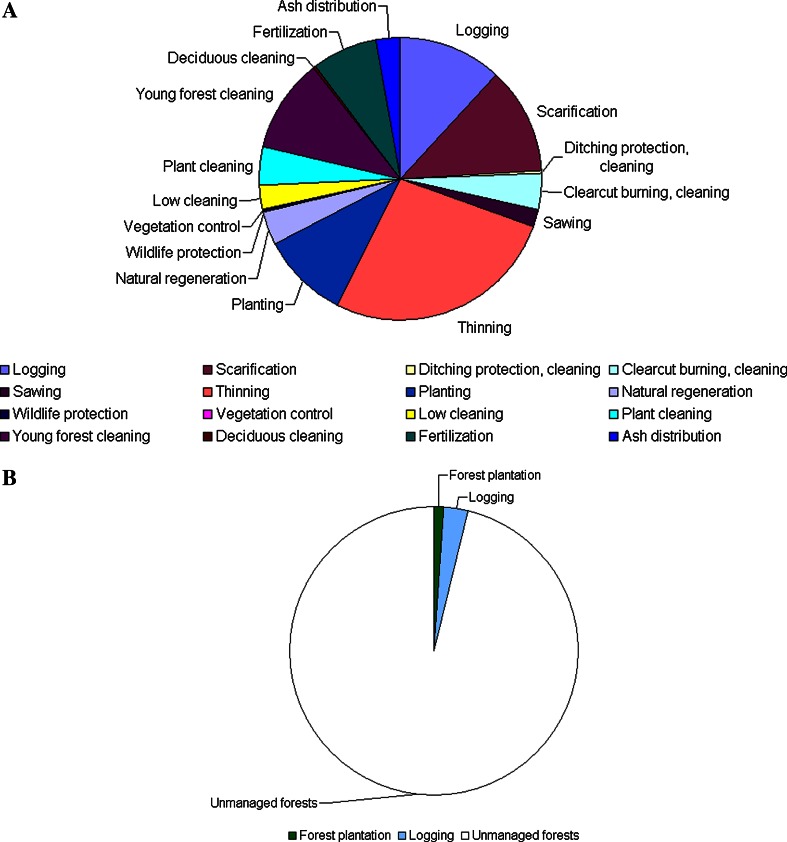
The area proportion (%) of different forest treatments of forests used for wood production from 2001 to 2010 in Bergslagen (Sweden) (**A**) and in Priluzje (Russia) (**B**) from 2006 to 2010. The total area was calculated only for those forest treatments which did not overlap with each other in space during the analyzed period

Silvicultural treatments (e.g., scarification, plantation, cleaning, thinning, fertilization, cleaning of undergrowth before felling and final felling) were identified for each planning unit within the tactical forest management plan for a period of 3–5 years. This information was used to plan road maintenance and construction, as well as silvicultural operations. Tactical planning was also needed to give the information to the company board, forestry and bioenergy industries about the amount and assortments of harvested wood that could be expected during the tactical planning period. The operational plan was developed for 1 year with the goal to identify the location of logging plots in order to estimate costs and to contract subcontractors. All information about the forest stands (including nature conservation values), forest roads, and market agreements for final felling were included into the tactical planning process. As an example, the diversity and area proportion of silvicultural treatments (in % from total area of forests used for wood production) in Bergslagen is shown in Fig. 2. In Bergslagen, treatments were applied on a cumulative area corresponding to 53 % of the total area in the period 2001–2010 (Table 3).

#### Russia-Priluzje

In Priluzje final felling was done by clear-felling methods. On average 0.5 % of the total area of forests used for wood production was subject to clear-felling annually (Table 3). The spatial distribution of harvested areas was concentrated mainly along the existing roads in Priluzje (Fig. 1). Plantation and commercial thinning was done on 0.2 % of the total area of forests used for wood production annually. After clear-felling the main approach in reforestation was to support natural regeneration by using seed trees and pre-established Norway spruce (Fig. 2). Thus the treatments were applied only on 0.7 % of the total area in the period 2006–2010 (Table 3).

## Discussion

### Different Interpretations and Implementations of the Sustained Yield Forestry Concept

Our results of our comparative study indicate that the current forest management regimes in both Bergslagen (Sweden) and Priluzje (Russia) were declared as being SY forestry. The principle for use of wood resources in both countries was that average annual harvests in the long run was equal to average annual net growth per year. Annual harvest in relation to annual net growth is perceived as a reliable and simple indicator of the sustainability of wood production. At the same time, the decision “harvest = growth” is a non-optimal solution. The best solution depends on, among other things, the age class distribution and the growth in different age classes. However, to switch from simple calculations and rules of thumb to a more advanced decision, requires (i) detailed and high accuracy data concerning the forest state, and (ii) an advanced forest Decision Support System.

The official definitions of the SY principle were expressed differently in two countries. In Sweden, a sustainable timber harvest level was presented as the main target of forest management, which can be interpreted as the perspective of the commercial company Sveaskog. By contrast, in Russia the official definition of AAC had a multi-stakeholder perspective, because it required “…multi-purpose, efficient, continuous and sustainable use of forests, according to the established age of final felling requirements for biodiversity conservation, maintenance of water protective and protective functions and other benefits of forests” (FAF 2011).

However, there was a clear discrepancy between implementation of SY forestry in the studied boreal FMUs when it comes to forest management planning and management practices (Table 2).

There is a profound difference in the approaches used to determine sustainable harvest levels. This originates from a different understanding of forest growth and yield in harvest scheduling. In Sweden a single tree growth model is used to understand the dynamics of existing forests, and the FMPP is a planning tool which allows implementing scientifically developed growth models in a real planning context. In Russia the main input data for estimation of sustainable harvest level comes from the inventory of standing wood volumes. The estimation of AAC is based on current status of forests and construction of forest dynamics based on stand age class distribution. To make projections of the future forest state without using a dynamic growth model, like for example the FMPP, the Russian AAC models are based on assumptions like “stands becomes 10 years older in 10 years”, “volume per hectare at a certain age will remain the same in the future as today”. We argue that these assumptions could lead to erroneous results in estimation of the sustainable harvest level. In support of this, the AAC for the Komi Republic decreased from 84 million m^3^ in 1950 to 26 million m^3^ in 2010. The discrepancy in AAC estimations can be attributed to the short time horizon used in Priluzje for AAC computation, which makes it more reflective of the time period’s specific forest age structure (and wood availability). Instead, the AAC in Russia could be seen as a plan for harvesting the existing stock of forest.

Additionally, in the Russian boreal biome the forest area used to estimate the sustainable harvest level is often larger than the area on which harvest is permitted. This occurs because the forests which are set aside according to the requirements of national FSC standard and not protected according to the national legislation are not excluded from the AAC calculation. Thus, the estimation of AAC is a strict formal exercise separated from other planning processes like identifying set-aside areas outside the national legislation. This error overestimates the real sustainable harvest potential. Moreover, natural disturbances such as wildfire are nearly absent in Bergslagen, but still prevalent in Priluzje. However, in Priluzje, even if the impact of these disturbances is implicitly considered when determining rotation based on experiences from naturally regenerated and unmanaged stands, due to reformation of the forest sector in the country major fire outbreaks has been hardly controlled. Thus, the whole idea of SY forestry could be void due to unexpected disturbance events. One would argue that, as a result, harvest rates are set at a higher level than what is actually available. On one hand, a higher harvest rate (or shorter rotation) encourages the harvest of a stand before a fire (or other disturbance agent) can destroy it (Reed 1984; Armstrong 2004). On the other hand, not considering the impact of stochastic events that influence AAC, particularly by changing the age structure, which has strong repercussions on the SY constraint, could lead to irreversible conditions. This includes both loss of economic opportunities, and the extirpation of wildlife populations such as demonstrated by Morgan et al. (2007) regarding the woodland caribou (*Rangifer tarandus caribou*) population of Labrador under an additive forestry/fire dynamics.

Furthermore, the term AAC has an annual resolution. Due to different natural disturbances or market fluctuations, the annual resolution of the long-term AAC does not necessarily guarantee sustainability of wood flow during a long period of time. In Sweden the tactical and operational forest management planning within the long sustainability horizon gives more opportunities to adapt harvests to business cycles for a “flexible harvest approach”, which supports an even flow of timber to the industry “for larger temporal unit instead of an annual resolution” (Hahn and Knoke 2010).

Implementation of SY forestry on the ground is different. The sustainable wood flow in Bergslagen is supported by a full range of silvicultural treatments. The FMPP optimizes the economic output from the forestry and it is translated to how to treat all stands with in an area for a rotation period. In Priluzje forest management is based on a custom of clearcutting with no or poor regeneration and most often driven by desire to get cheap wood (Carlsson 2000). Hence, forestry in Priluzje can be characterized as wood mining in the sense that a minimum of silviculture measures are applied, stands are regenerating naturally, and the harvest level is adapted to the regeneration period. Unless AAC is reduced, this is likely to sooner or later lead to a timber shortage, i.e., a sharp decline in the AAC. This could to some extent be avoided by the introduction of more intensive silvicultural measures, which requires an appropriate road network.

To conclude, while the term SY forestry is used in both Sweden and Russia, it obviously has different interpretations: from forestry based on natural regeneration with minimum investments in silviculture in Russia to maximum yield forestry based on high-input forest management in Sweden. Our study shows that it is challenging to make meaningful comparisons of value-laden concepts between countries and FMUs with different management histories, policies, and infrastructure. However, we assume that the main reason behind the observed difference is linked to the fact that in Sweden SY forestry is driven by market economy and based on economic principles; whereas in Russia SY forestry is very extensive, trying to balance opportunistic wood provisions from nature along a mid-term horizon in respect with biological growth.

### SY Forestry and Sustainable Forest Management: How Much of What?

From an economic perspective the forest in Bergslagen is much more valuable for wood production than the forest in Priluzje, measured on either per cubic meter or per hectare basis. The reasons include a well-developed and dense road network, proximity to consumers and export markets, and relatively long history of high-input forest management with the goal of maximizing the productivity of the forest measured in terms of charcoal in the past, then sawlogs and pulpwood, and now also biomass for energy (Angelstam et al. 2011a). The resulting sustained wood production is an outcome of the classic German-school forest management approach (Puettmann et al. 2008). The forest management strategy in Bergslagen is largely about managing forest growth, and with some considerations to ecological criteria. The result is a forest dominated by relatively young even-aged stands and the break-up of forest tracts into many smaller, widely dispersed operational units, with their own optimized silvicultural schedule trajectories. By contrast, Priluzje has a both a shorter history and low-input forest management focused on sawlog and pulpwood harvest. The road network is far from fully developed, and cannot be used during all seasons. The economically rational way of using this forest is to proceed as to manage the forest using relatively large harvest blocks to minimize the per cubic meter cost of road network development. The forest management strategy in Priluzje can be characterized as rationing of the existing stock of timber. The private forest companies and state forest administrations make very limited efforts to maintain or improve the wood resources through silvicultural treatments, and have not made investments in development of forest transport infrastructure during the last decade. As a consequence, there is a lack of sawlog and pulpwood from Norway spruce and Scots pine in the Komi Republic to meet the demand from the forest industry, and instead an abundance of pulpwood from deciduous trees. At present in Priluzje the AAC consists of wood mainly from deciduous trees (60 %) and the rest from conifers (40 %) (CF 2008). The distribution of wood stock and tree species composition shows that in 20–50 years the share of deciduous trees could increase up to 70–80 % in AAC (CF 2008). Additionally, as a result of undeveloped forest transport infrastructure forests are systematically over-harvested locally, mainly along important transport routes (Carlsson 2000). Therefore, we conclude that from an economic perspective the forest mining approach to SY forestry as it is implemented in Priluzje does not sustain the resource base that is currently required by the existing forest industries.

From an ecological perspective, a long history of maximum SY forestry (Ek 1995; Angelstam et al. 2011a) in Bergslagen has transferred the once naturally dynamic forests to an efficient wood production system. This has resulted in a loss of compositional, structural, and functional elements of biodiversity found in naturally dynamic landscapes (Bütler et al. 2004), and there are no large intact forest areas left (Elbakidze et al. 2011). As Trosper (2003) noted: “High production of timber inevitably raises questions about sustaining that yield and about the condition of forest in general. If the yield of one resource is driven too high, the yield of other uses fall, and the future yield of the dominant resource is also threatened”.

**Fig. 3:**
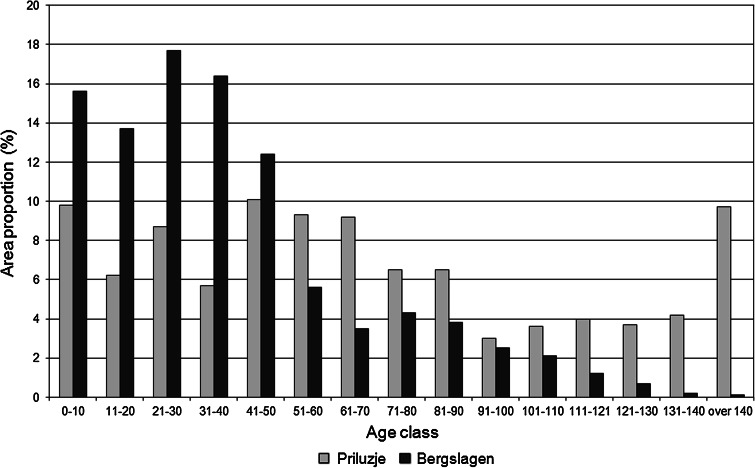
Age structure of forests in Bergslagen (Sweden) and Priluzje (Russia)

The comparison of age structure of forests used for wood production in Bergslagen and Priluzje shows that there is no biologically old forest left in the Swedish FMU. By contrast in Priluzje forest stands of different ages are present, and with quite large areas of forests older than 140 years (Fig. 3). The tree species composition has been changed considerably in Bergslagen because the volumes of tree species such as birch and aspen, which have been less desirable by the market, have been actively reduced. Today, however, there are attempts to increase the proportion of deciduous trees to promote biological diversity and to provide the industry with hardwood timber (Sveaskog 2012) On the contrary, in Priluzje birch and aspen are abundant due to a lack of active coniferous forest regeneration and pre-commercial thinning. Additionally, the systematic silvicultural treatments has left no space for natural disturbances such as wildfire and insect outbreaks in Bergslagen, while in Priluzje such processes are quite common. This presents Priluzje with a real advantage for biodiversity conservation that can occur with a lower opportunity cost than in Bergslagen. Additionally, the opportunities for success are likely much greater due to Priluzje’s older age class structure, and larger contiguous blocks of formally and voluntarily protected areas (Elbakidze et al. 2011) if measures are taken now. Thus, we conclude that from ecological perspective, the current situation for biodiversity conservation is more favorable in Priluzje than in Bergslagen.

From a social point of view both Bergslagen and Priluzje are faced with challenges related to rural development. In Bergslagen effective mechanization has severely reduced the number of forest workers required for forest management. To compensate for this loss, recreation and tourism are emerging businesses (Andersson et al. 2012). In Russia during the Soviet time, forestry had very low nominal transport costs, and access to governmental subsidies for development not only of industrial infrastructure but also for the costs of social infrastructure such as villages, housing, kindergartens, and public transportation (Madison 1968). To cope with the disappearance of this support to rural development, in Priluzje forest set-asides were thus made to provide local communities with forest for local use near villages.

The meaning and implementation of SY forestry in both countries provoke continuous public debate and concerns (Beland Lindahl and Westholm 2011; Sandström et al. 2011). In Sweden there are concerns among stakeholders outside the forest sector about the negative impact of intensive forest management on forest landscapes linked to a long history of maximum SY forestry. This applies to forest ecosystem integrity and biodiversity conservation (Angelstam et al. 2011a; Elbakidze et al. 2011) as well as rural development (Bostedt and Mattsson 1995), and to cultural forest values (Zaremba 2012).

In the Russian Federation the main concern among foresters is about insufficient management of forest resources, which does not provide sustainable economic outcomes for forest industry. This ambition is actually creating challenges for nature conservation and rural development. For example, large regions of the Russian boreal forests are severely affected by an accelerated harvesting of wood during the last decades (Elbakidze et al. 2007), and the intact forest landscapes are shrinking by 3 % annually due to logging (Mayer et al. 2005). There are also discussions that forestry in the shape of forest mining should not allow to be certified according to the national FSC standard (A. Yaroshenko, pers. comm.).

Does SY forestry correspond to SFM policy? It depends on how it is implemented. We agree with Vincent and Binkley (1993) that:

Optimal management will tend toward dominant use in each stand whenever one of the two products produced by the forest is more responsive to the management efforts than is the other. The dominant use can be superior to multiple use even when the stands in a forest are identical; and the strength of the tendency toward dominant use is linked to the rate at which returns to management efforts diminish.

The answer to the question about how SY forestry contributes to SFM depends on the context. Maximum sustained wood yield as the sole management paradigm has probably run its course. The history and subsequent characteristics of the forest of concern, as well as the suite of economic, ecological, social, and cultural goals of the people affected by the forest all matter. This has varied and will vary across time and space.

SY forestry focuses on wood resources as a commodity and its efficient and sustainable utilization. This type of sustainability is traditionally based on command-and-control thinking and execution (Holling et al. 1998), and requires that forest ecosystem behavior is predicted based on the assumption that ecosystem productivity is stable, natural disturbances are controlled, and ecosystem outputs are stabilized (Clapp 1998; Folke et al. 2003). Based on historical evidence, we agree that without a command-and-control perspective in forestry it would not have been possible to derive the level of economic benefits from forests as it has.

However, SFM considers a broad range of perspectives including ecological, economic (also including other then wood production), and social–cultural. This is based on the assumption that a managed forest is a complex system with specific attributes such as nonlinearity, uncertainty, emergence, scale, and self-organization (Berkes et al. 2003). Thus, to implement SFM on the ground: “it may be worthwhile to remove sustainability constraints on timber volumes and replace them with sustainability constraints on non-timber resources that suffer from irreversibility, thresholds, public good characteristics of resources and incomplete or absent property rights” (Luckert and Williamson 2004). Under this scenario, the concept of SY forestry only could disappear as a separate concept, and sustainable wood production will be just one criterion to be considered in SFM along with other criteria. In other words, policies would need to be changed so that maximum SY across entire FMUs will no longer be the only explicit goal of SFM.

## Electronic supplementary material

Below is the link to the electronic supplementary material.

Supplementary material 1 (PDF 17 kb)
