# Supplementary material for: Sustained Yield Forestry in Sweden and Russia: How Does it Correspond to Sustainable Forest Management Policy?
Source: Ambio. 2013 Mar 10;42(2):160–73. doi: 10.1007/s13280-012-0370-6 (PMC3593033; doi:10.1007/s13280-012-0370-6)
Supplement: Supplementary file 1 — Supplementary material 1 (PDF 17 kb) [file 13280_2012_370_MOESM1_ESM.pdf]

Electronic Supplementary Material

Sustained yield forestry in Sweden and Russia: How does it correspond to sustainable forest management policy?

Marine Elbakidze, Kjell Andersson, Per Angelstam, Glen W. Armstrong, Robert Axelsson, Frederik Doyon, Martin Hermansson, Jonas Jacobsson, Yurij Pautov

Table S1 Formulas for calculation of annual allowable cut in the Russian Federation according to the regulation of the Federal Agency of Forestry from 2011

	Formula for calculation of annual allowable cut:	
	By area, ha	By volume, m^3 ha^{-1}
Even-age is used when forests have relatively even distribution of forest stands and timber resources in different age classes	$Lp = F/U$	$Vp= Lp\ V_{m+ov}$
The first age is used when forests have depleted wood stock in mature and over-mature forest stands (less than 20% from the total growing stock)	$L_1= F_{pm} + F_{m+om}/2K$	$V_1 = L_1\ V_{m+ov}$
The second age is used when the wood stock of mature and over mature forest stands correspond to more than 50% of the total growing stock	$L_2= F_{md} + F_{pm} + F_{m+om}/2K$	$V_2= L_2\ V_{m+ov}$
Integral is used in the same cases as the previous one (the second age)	$Lint = (0.2F_y + 0.6F_m + F^2_m + 1.4F_{pm} + 1.8F_{m+ov}) \times 0.01$	$Vint= L\ int\ V_{m+ov}$

F – the total area of forests used for wood production; U – age of final felling; F_y – an area of young forests; F_{md} – an area of middle age forests; F_{pm} – an area of pre-mature forests; F_m – an area of mature forests; F_{ov} – an area of over mature forests; K – a duration of age class in years; V_{m+ov} – mean volume of wood per hectare in mature and over-mature forests.
